# Alcohol Endotheliitis: A Report of a Rare Case

**DOI:** 10.7759/cureus.62822

**Published:** 2024-06-21

**Authors:** Deepaswi Bhavsar, Khushboo Goyal, Renu Magdum, Iqra Mushtaq, Shreya Gandhi

**Affiliations:** 1 Ophthalmology, Dr. D. Y. Patil Medical College, Hospital and Research Centre, Pune, IND

**Keywords:** endothelial pump, toxic endothelitis, topical steroids, corneal edema, alcohol

## Abstract

Sudden transient loss of vision after an acute bout of alcohol consumption in patients with chronic alcoholism is rare. The underlying mechanism is a transient depression of the endothelial pump due to ethanol toxicity following a large amount of alcohol consumption in chronic alcoholic patients. Here, we report a rare case of a 60-year-old male patient who came to the outpatient department with complaints of sudden loss of vision associated with redness following a large amount of alcohol consumption. The case was managed by prompt diagnosis and topical and oral corticosteroid therapy. This is a rare case of acute toxic endotheliitis due to ethanol toxicity with only a few cases reported in the past.

## Introduction

The corneal endothelium is one of the important factors in maintaining corneal transparency by keeping the cornea in a relatively dehydrated state with the help of an endothelial pump. Any kind of inflammation in the corneal endothelium will lead to endotheliitis which subsequently leads to vision loss. Causes of endotheliitis include viral (most common), fungal, bacterial, drug-related, procedure-related, environmental, toxic, etc. [1]. The exact etiology of corneal edema is not known but it is presumed to be due to a transient depression in the endothelial pump activity which is caused by ethanol [2]. Ethanol and its metabolite acetaldehyde are presumed to cause toxic morphological and biochemical effects to the corneal endothelium and also to the stromal proteins [2].

We report a case of toxic endotheliitis due to ethanol poisoning after an episode of acute alcohol consumption in chronic alcoholic patients.

## Case presentation

A 60-year-old alcoholic male came to the ophthalmology outpatient department with complaints of sudden diminution of vision associated with redness in both eyes. The patient gives a history of acute alcohol consumption in large amounts of local liquor approximately 500 to 525 ml one day back. There was no history of trauma or any history of similar attacks in the past. He had no known systemic illness. The patient has been a chronic alcoholic for the past 30 years with daily consumption of 30 to 60 ml of alcohol. The best corrected visual acuity in the right eye was 6\24 and that in the left eye was 1\60.

**Figure 1:**
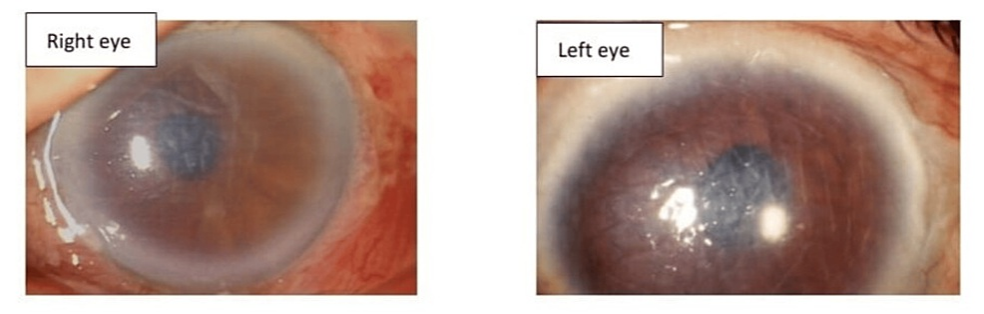
Anterior segment photo of both eyes showing corneal edema and Descemet membrane folds at the time of presentation

On examination, both eyes had evidence of diffuse conjunctival congestion. Corneal edema was present with dense stromal haze all over. Descemet membrane folds were present diffusely. The anterior chamber (AC) shows the reaction in both eyes with +3 cells and grade 2 flare. Iris and pupil were hazily seen but appeared within normal limits. The AC angle was wide open in both eyes according to Van Herick's grading (grade 4). Both eyes show grade 1 nuclear sclerosis in the lens (Figure 1). Fundus examination with direct and indirect ophthalmoscopy was tried but the retina was not visualized due to hazy media. On the B scan, the posterior segment was within normal limits with no evident edema of the optic nerve head.

**Figure 2:**
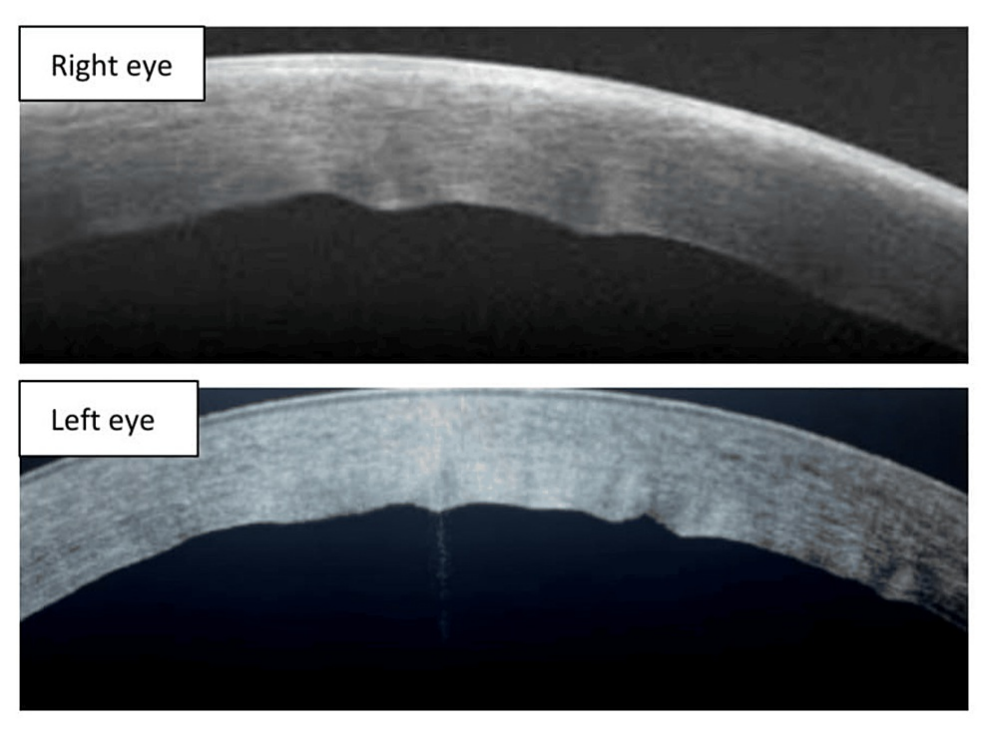
Anterior segment optical coherence tomography photo of both eyes showing corneal edema with Descemet membrane folds at the time of presentation

On anterior segment optical coherence tomography (AS-OCT), there was increased thickness of the cornea with Descemet membrane folds present in both eyes (Figure 2). The central corneal thickness as measured was 630 microns in the right eye and 640 microns in the left eye.

The patient was diagnosed with alcohol-induced acute toxic endotheliitis. A differential diagnosis of viral endotheliitis was considered. However, the condition being bilateral and diffuse corneal involvement was against viral etiology. His complete blood cell counts were within normal limits ruling out an infectious etiology. He was started on steroids. Topically, eyedrop prednisolone acetate 1% was given twice hourly with eyedrop homatropine 2% at bedtime in both eyes. He was also started on the tablet Wysolone 60 mg (oral steroids) once a day.

**Figure 3:**
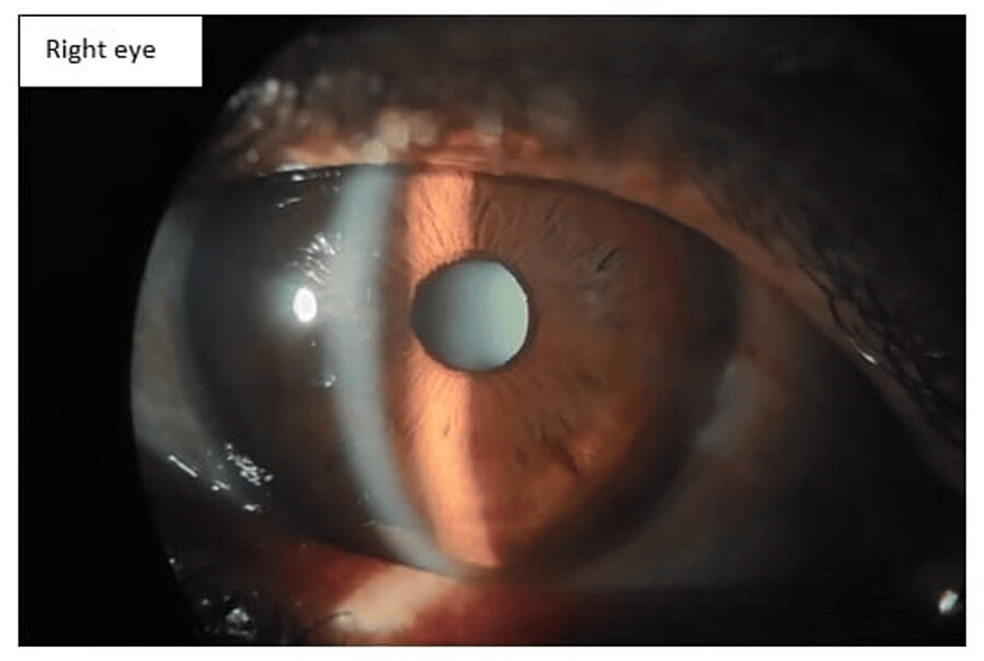
Anterior segment photo of the right eye after six weeks of treatment showing resolved corneal edema

**Figure 4:**
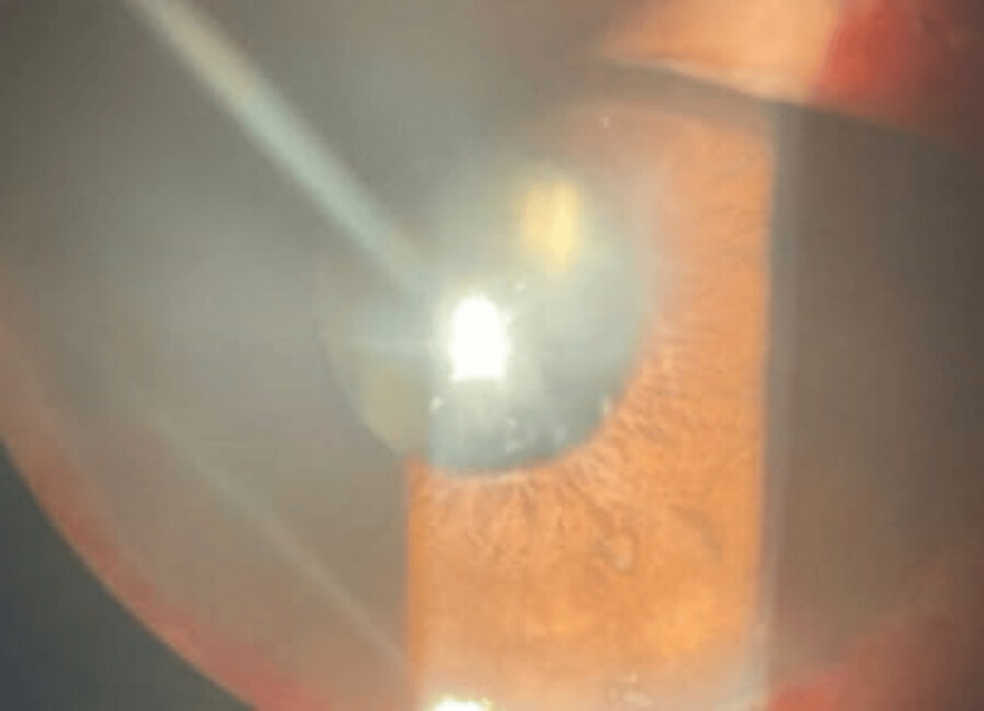
Anterior segment photo of the left eye after six weeks of treatment showing resolved corneal edema

The patient was followed up after two weeks. On slit-lamp examination, conjunctival congestion was markedly reduced. Corneal edema subsided and stromal haze was reduced. Minimal Descemet membrane folds were present. AC reaction subsided with no evidence of cells and flare. The patient’s steroid drop was tapered to six times a day. Tablet Wysolone was tapered to 50 mg and tapering was advised every five days. At the end of four weeks, the cornea was clear with minimal stromal haze present in the left eye and the right eye was within normal limits. Topical steroid drop was tapered four times and tapering was advised every five days. At the end of six weeks, the patient recovered completely with a vision of 6\12 in the right eye and 6\24 in the left eye with a normal anterior segment (Figures 3, 4).

**Figure 5:**
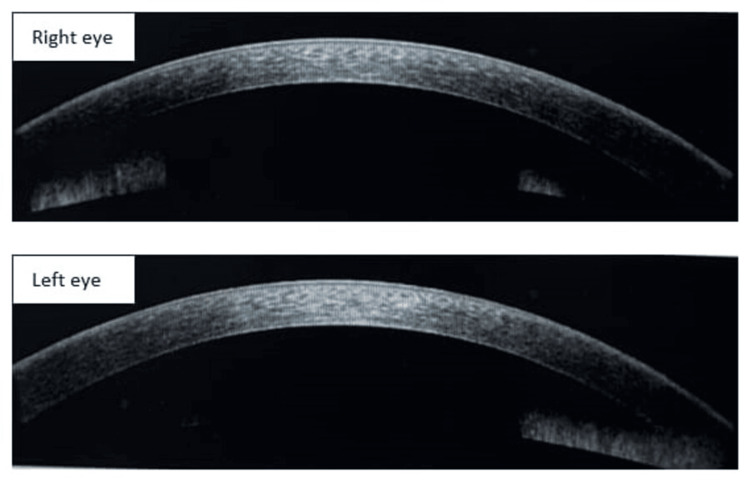
Anterior segment optical coherence tomography photo of both eyes showing resolved corneal edema after treatment of six weeks

AS-OCT of both eyes was normal with resolved corneal edema (Figure 5). The central corneal thickness as measured was 520 microns in the right eye and 540 microns in the left eye.

## Discussion

The above case is a rare presentation of bilateral corneal edema following an acute bout of alcohol consumption in chronic alcoholic patients. Toxic endotheliitis following ethanol toxicity is rare. Only a few cases were reported in the past. The mechanism involved is temporary suppression of endothelial pump activity which is supposed to be due to oxidative damage to endothelium stromal proteins by ethanol and aldehydes [2]. In endotheliitis, the patient presents with corneal edema, stromal haze, and Descemet membrane folds. Along with that keratic precipitates on endothelium and AC reaction is present commonly. Limbal ingestion and vascularization may also be present rarely [1].

Vision loss secondary to optic nerve head toxicity due to methanol poisoning is commonly seen in chronic alcoholic patients. Methanol is cheap and readily available. Hence, it is frequently adulterated in alcoholic beverages. Methanol and moreover its metabolite formic acid is responsible for toxic effects [3]. Our case is rare as vision loss is due to endothelial pump suppression due to ethanol toxicity leading to corneal edema.

Shiono et al. first reported corneal edema after intake of alcohol due to toxicity. Before that, alcohol was presumed to cause optic nerve head toxicity only [4]. In Ranjan et al. study of acute toxic endotheliitis following alcohol consumption, they have given IV steroids in addition to topical steroids, while in our study, IV steroid administration was not done [2].

## Conclusions

Bilateral toxic endotheliitis following a large amount of alcohol consumption in chronic alcoholics is a rare presentation of endotheliitis. It is a reversible condition as vision loss is transient. Timely diagnosis and prompt treatment are the key. Other causes of sudden vision loss should be ruled out.

## References

[1] Moshirfar M, Murri MS, Shah TJ (2019). A review of corneal Endotheliitis and endotheliopathy: differential diagnosis, evaluation, and treatment. Ophthalmol Ther.

[2] Ranjan A, Murthy SI, Rathi VM, Sangwan VS (2018). Acute bilateral toxic endotheliitis following alcohol consumption. Ocul Immunol Inflamm.

[3] Rathi M, Sakhuja V, Jha V (2006). Visual blurring and metabolic acidosis after ingestion of bootlegged alcohol. Hemodial Int.

[4] Shiono T, Asano Y, Hashimoto T, Mizuno K (1987). Temporary corneal oedema after acute intake of alcohol. Br J Ophthalmol.

